# Mettl14 inhibits bladder TIC self-renewal and bladder tumorigenesis through *N*^6^-methyladenosine of Notch1

**DOI:** 10.1186/s12943-019-1084-1

**Published:** 2019-11-25

**Authors:** Chaohui Gu, Zhiyu Wang, Naichun Zhou, Guanru Li, Yiping Kou, Yang Luo, Yidi Wang, Jinjian Yang, Fengyan Tian

**Affiliations:** 1 Department of Urology and Henan Institute of Urology, Zhengzhou Key Laboratory for Molecular Biology of Urological Tumor Research, The First Affiliated Hospital of Zhengzhou University, Zhengzhou, Henan 450052 People’s Republic of China; 2 Department of Pediatrics, The First Affiliated Hospital of Zhengzhou University, Zhengzhou, Henan 450052 People’s Republic of China

**Keywords:** *N*^6^-methyladenosine, Bladder tumorigenesis, Bladder TIC, Notch1, Mettl14, Self-renewal

## Abstract

**Background:**

*N*^6^-methyladenosine (m^6^A) emerges as one of the most important modification of RNA. Bladder cancer is a common cancer type in developed countries, and hundreds of thousands of bladder cancer patients die every year.

**Materials and methods:**

There are various cells in bladder tumor bulk, and a small population cells defined as tumor initiating cells (TIC) have self-renewal and differentiation capacities. Bladder TICs drive bladder tumorigenesis and metastasis, and their activities are fine regulated. However, the role of *N*^6^-methyladenosine in bladder TIC self-renewal is unknown.

**Results:**

Here, we found a decrease of *N*^6^-methyladenosine in bladder tumors and bladder TICs. *N*^6^-methyladenosine levels are related to clinical severity and outcome. Mettl14 is lowly expressed in bladder cancer and bladder TICs. Mettl14 knockout promotes the proliferation, self-renewal, metastasis and tumor initiating capacity of bladder TICs, and Mettl14 overexpression exerts an opposite role. Mettl14 and m^6^A modification participate in the RNA stability of Notch1 mRNA. Notch1 m^6^A modification inhibits its RNA stability. Notch1 plays an essential role in bladder tumorigenesis and bladder TIC self-renewal.

**Conclusion:**

This work reveals a novel role of Mettl14 and *N*^6^-methyladenosine in bladder tumorigenesis and bladder TICs, adding new layers for bladder TIC regulation and *N*^6^-methyladenosine function.

## Introduction

Bladder cancer is a serious cancer in the world, especially in advanced countries [1]. There are many kinds of cells in bladder tumor, including bladder cancer stem cells (CSC), or tumor initiating cells (TIC) [2]. Bladder TICs, a small population of cells in bladder tumors, have self-renewal, differentiation and tumor-initiating capacities [2]. Recently, increasing markers of bladder TICs were identified, and CD44 is one of the most widely-accepted markers [3]. Compared with CD44^−^ cells, CD44^+^ cells show enhanced self-renewal and tumor-initiating capacities. Bladder TICs escape anoikis and initiate oncospheres in FBS-free medium, but bladder non-TICs can’t survive [4]. Accordingly, sphere formation assay emerges as one of the most important assays to detect bladder TIC self-renewal. Besides sphere formation, transwell invasion assay can also be used for bladder TICs because of the critical role of bladder TICs in bladder metastasis and invasion [2, 5]. Like TICs in many other tumors, bladder TICs harbor enhanced tumor-initiating capacities, which can be examined by gradient tumor initiating assay [6–8]. Highly expressing ABCG2 and other pump molecules, TICs are resistant to drug treatment [9]. Despite of the critical role of bladder TICs in bladder tumor formation, metastasis, drug resistance and recurrence, the biological characteristics of bladder TICs are largely unknown.

Like TICs in other tumors, bladder TICs are fine regulated, and the precise regulations of bladder TIC self-renewal are still largely unknown [10, 11]. *N*^6^-methyladenosine (m^6^A) is the most abundant modification of mRNA in human and mice, and is very conserved among species [12]. m^6^A modification is identified in plants, yeast, insects, virus and so on. Recently, m^6^A modification was also found on some non-coding RNAs, including tRNA, rRNA, lncRNA and snRNA [13]. m^6^A modification is reversible, which is added by methyltransferase complex and removed by m^6^A demethylases. m^6^A methyltransferase complex is comprised of METTL3, METTL4, METTL14, WTAP, VIRMA and so on [14, 15]. On the contrary, FTO and AlkBH5 induce m^6^A demethylases [16, 17]. The influence of m^6^A in RNA stability is dependent on m^6^A readers, including YTHDF1, YTHDF2, YTHDF3 and YTHDC1 [18]. m^6^A modification participates in many biological processes, including spermatogenesis and embryonic development [19–21], circadian period [22], DNA damage [23], hematopoietic stem cells [24] and tissue homeostasis [25]. As for tumor biology, m^6^A modification exerts its role in tumorigenesis, proliferation and metastasis. Mettl14 is lowly expressed in hematopoietic stem cells and liver tumor cells. m^6^A methyltransferase Mettl14 attenuates the tumorigenesis of AML [26]. Recently the inhibitory role of Mettl14 in liver tumorigenesis and metastasis was also revealed. Demethylase FTO drives tumorigenesis of acute myeloid leukemia [27]. Another demethylase, ALKBH5 also exerts an oncogenic role in glioblastoma tumorigenesis [11]. However, the roles of *N*^6^-methyladenosine and related enzymes in bladder tumorigenesis and bladder TIC self-renewal are largely unknown.

Here, we found that m^6^A modification and Mettl14 were lowly expressed in bladder tumorigenesis and bladder TICs. m^6^A level and Mettl14 expression were negatively related to the bladder cancer severity and clinical outcome. m^6^A modification and Mettl14 inhibited bladder tumorigenesis and bladder TIC self-renewal through Notch1 signaling, adding a new layer of bladder TIC regulation and m^6^A function.

## Materials and methods

### Reagents and samples

Anti-β-actin (cat. no. A1978) and DAPI (cat. no. 28718–90-3) were obtained from Sigma-Aldrich. Anti-m^6^A antibody was purchased from Synaptic Systems. Anti-Mettl14 (ab220030) antibody and m6A quantification kit (ab185912) were from Abcam. Fluorescence-conjugated secondary antibodies were obtained from Molecular Probes Life Technologies.

Primary bladder cancer samples were obtained from the Departments of Urology and Henan Institute of Urology, The First Affiliated Hospital of Zhengzhou University with informed consent. The details for bladder tumors used in this work were: #1, early bladder cancer, 63 years old, female, tumor size, 23.1 × 17.3 × 16.2 mm, non-invasive, stage I, non-metastasis. #2, advanced bladder cancer, 72 years old, male, tumor size, 38.3 × 21.8 × 19.9 mm, invasive, stage IV, metastasis. #3, advanced bladder cancer, 69 years old, female, tumor size, 35.5 × 26.8 × 21.9 mm, invasive, stage III, non-metastasis. #4, early bladder cancer, 78 years old, male, tumor size, 29.2 × 18.9 × 17.2 mm, non-invasive, stage II, non-metastasis. #5, advanced bladder cancer, 75 years old, male, tumor size, 31.9 × 29.1 × 19.8 mm, non-invasive, stage II, non-metastasis #6. advanced bladder cancer, 73 years old, male, tumor size, 31.3 × 28.8 × 28.1 mm, non-invasive, stage II, non-metastasis.

### Primary cell isolation and culture

Primary bladder cancer cells were obtained from bladder cancer patients. For primary cell isolation, a portion of excised tumor was incubated in Hanks balanced salt solution (HBSS; Gibco) and transported quickly to the laboratory on ice. Then the samples were cut into small fragments, and digested in HBSS containing 0.1% type I collagenase, 0.05% type IV collagenase, 0.03% pronase, and 0.01% deoxyribonuclease at 37 °C for 30 min. The sample was filtered through 70-μm-nylon filter and centrifuged for 4 min at 50 x g in 4 °C. Bladder cancer primary cells were in precipitation, and cell survival & purification were examined. For bladder TIC enrichment, bladder cancer primary cells were incubated with CD44 antibody for FACS, and TICs were used for sphere formation assay, m6A detection and other assays.

### Sphere formation

Sphere formation assay was detected to examine the self-renewal of bladder TICs. 500 CD44^+^ bladder TICs were cultured in FBS-free DMEM/F12 medium (supplemented with 1 × B27 supplement, 1 × N2 supplement, 20 ng/ml bFGF and 20 ng/ml EGF) and seeded into Corning® Costar® Ultra-Low Attachment Multiple Well Plate (cat. no. 3471, Corning). Two weeks later, sphere images were taken and sphere numbers were counted. For cell line detection, 1000 T24 cells were used for sphere formation.

### CRISPR/Cas9 knockout

*Mettl14* and *Notch1* knockout cells were constructed through CRISPR/Cas9 approach. For knockout, sgRNAs were designed according to online tool (http://crispr.mit.edu/) and cloned into lentiCRISPRv2, which were transfected into 293 T cells for lentivirus package, and the lentivirus was concentrated with PEG-it Virus Precipitation Solution (System Biosciences). Bladder cancer primary cells were infected with lentivirus, and the transfected cells were collected by puromycin selection. Knockout efficiency was confirmed by Western blot, and then used for sphere formation assay, transwell invasion assay or other functional assays.

### Immunohistochemistry

For immunohistochemistry, 5-μm bladder cancer sections were treated with xylene (10 min × 2), 100% alcohol (5 min × 2), 95% alcohol (5 min), 75% alcohol (5 min), PBS (5 min), 3% H2O2 (20 min) and then boiled in Tris/EDTA buffer (PH 9.0) for antigen retrieval (20 min). Then the samples were incubated with anti-m^6^A (1:500 dilution in PBS) or anti-Mettl14 (1:500 dilution in PBS) antibodies for 2 h. After washing three times, HRP-conjugated secondary (1:500 dilution in PBS) and 3,3′-diaminobenzidine were used for visualization.

### Dot blot

For m^6^A dot blot, RNA were extracted from bladder tumor, TICs and spheres using standard Trizol method, and then spotted onto nylon membrane. The samples were crosslink with UV treatment, and followed by m^6^A antibody incubation (1:2000 dilution in PBS, supplemented with 5% milk) and subsequent HRP-conjugated secondary antibody (1:5000 dilution in PBS, supplemented with 5% milk), finally the samples were detected with 3,3′-diaminobenzidine. For loading control, 0.02% methylene blue was used to stain the same RNA samples.

### FACS

For FACS sorting or detection, samples were incubated with Phycoerythrin (PE)-conjugated CD133 (1:300 dilution in FACS buffer) or control antibodies (1:300 dilution in FACS buffer) for 30 min on ice, and then subjected to FACS. For FACS sorting, CD133^+^ bladder TICs and CD133^−^ non-TICs were enriched. For detection, FlowJo software (FlowJo v10) was used for data analyses.

### Statistical methods

For bladder TIC ratio analysis, 10, 1 × 10^2^, 1 × 10^3^, 1 × 10^4^ and 1 × 10^5^ cells were injected into BALB/c nude mice for three months’ tumor formation. The ratios of bladder TICs were calculated by ELDA (extreme limiting dilution analysis) with online software (http://bioinf.wehi.edu.au/software/elda/). For most experiments, two tailed unpaired Student’s t-test was used for statistical analysis.

## Results

### Decreased content of m^6^A modification in bladder cancer

**Fig. 1:**
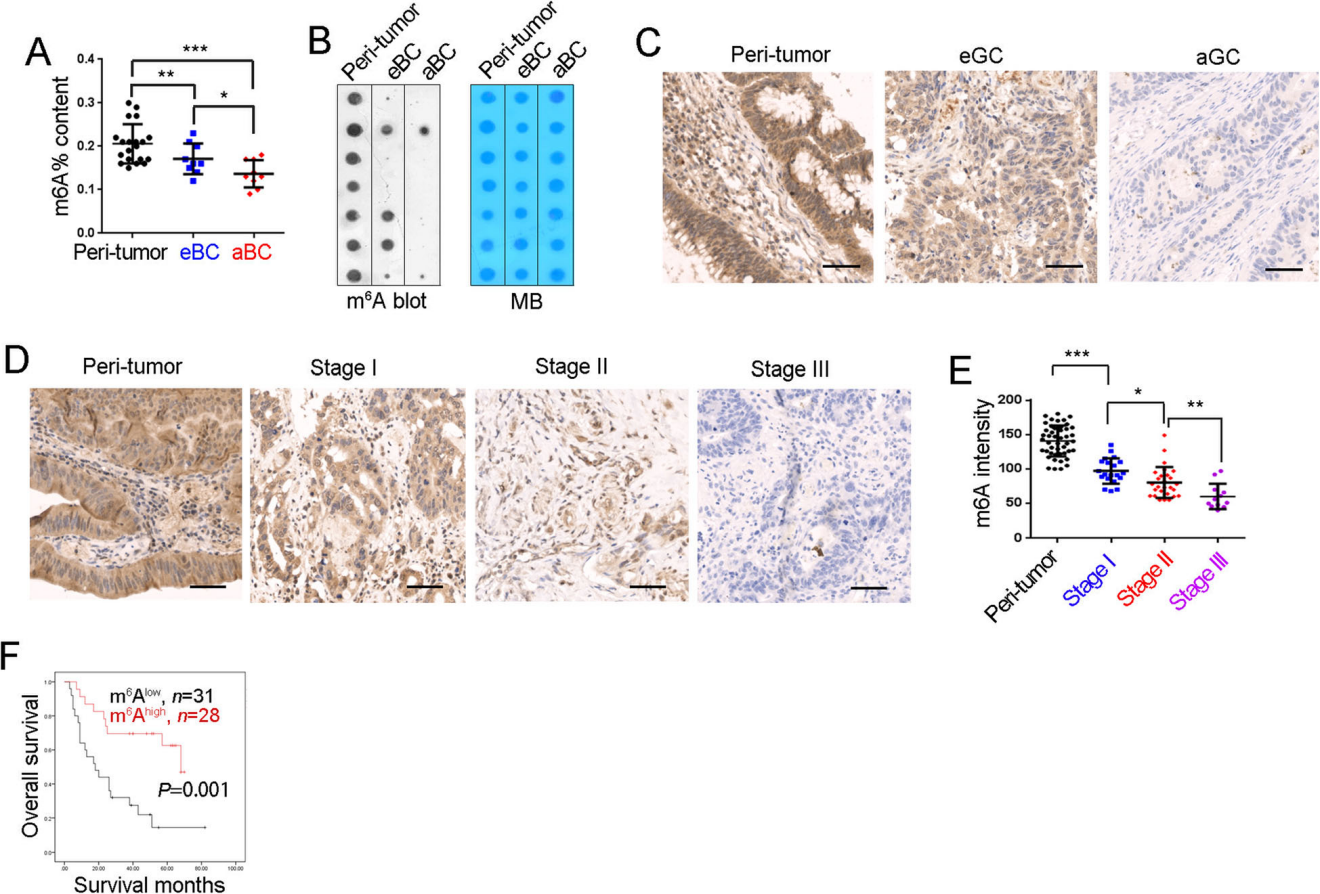
Decreased content of m^6^A modification in bladder cancer. **a** m^6^A modification levels in 20 peri-tumors, 10 early bladder cancer samples (eBC) and 20 advanced bladder cancer samples (aBC) were examined with m6A quantification kit. **b** m^6^A modification in peri-tumors, eBC and aBC was examined by Dot blot analyses. Representative results of 7 peri-tumors, 7 eBC and 7 aBC samples were shown. **c** Immunohistochemistry of m^6^A antibody. 20 peri-tumors, 10 eBC cancer and 10 aBC were used for immunohistochemistry, with similar results. **d**, **e** m^6^A was detected using tissue array containing 46 peri-tumors, 20 stage I, 27 stage II and 12 stage III bladder tumors. Typical images were shown in D and quantitative results were shown in **e**. **f** Bladder cancer samples were grouped into two subsets according to m^6^A levels, and Kaplan–Meier survival analysis was performed. *P < 0.05, **P < 0.01, ***P < 0.001, by two-tailed T test. At least three independent experiments were performed and got similar results

As the most widely distributed RNA modification in mammalian cells, m^6^A modification exerts critical roles in many biological processes. However, its role in bladder tumorigenesis and bladder TICs is unknown. In this work, we focused on the role of m^6^A modification in bladder tumorigenesis and bladder TICs, and we detected the content of m^6^A modification in bladder tumor first.

m^6^A modification was detected in non-tumor, early and advanced bladder tumors, and decreased m^6^A content was observed along with bladder tumorigenesis. Moreover, m^6^A modification was related to clinical severity (Fig. 1a). The reduction of m^6^A modification in bladder cancer was also validated by RNA dot blot (Fig. 1b), immunohistochemistry (Fig. 1c) and bladder cancer tissue array (Fig. 1d, e). As expected, lower content of m^6^A modification was also detected in advanced tumors by bladder cancer tissue array and immunohistochemistry (Fig. 1e). Moreover, m^6^A modification was also related to the clinical outcome of bladder tumor patients (Fig. 1f). Taken together, m^6^A modification content was lower in bladder tumors and related to clinical severity.

### Decreased m^6^A modification in bladder TICs

**Fig. 2:**
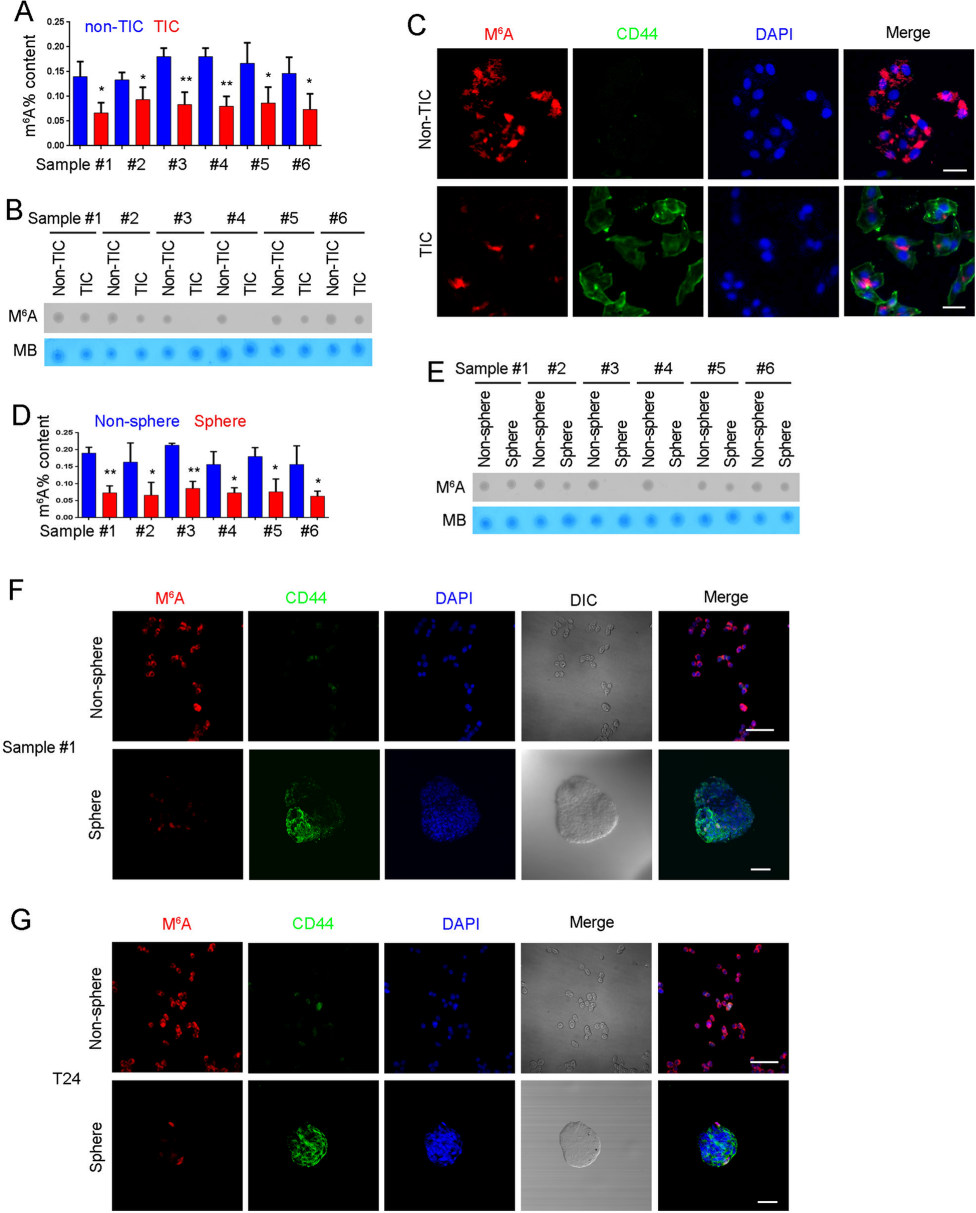
m^6^A modification was reduced in bladder TICs. **a** Bladder TICs and non-TICs were sorted by FACS with CD44 antibody, and mRNA was extracted for m^6^A detection. Six bladder tumors were used for TIC enrichment and subsequent m^6^A detection. **b** m^6^A RNA dot blot in bladder TICs and non-TICs. RNA extracted from bladder TICs and non-TICs was examined for m^6^A modification. Six samples were examined and got similar results. **c** FACS enriched TICs and non-TICs were stained with m^6^A and CD44 antibodies, and visualized by confocal microscopy. **d** Sphere formation was performed, followed by m^6^A detection using spheres and non-spheres. **e** Spheres and non-spheres were generated from bladder primary cells and m^6^A modification was detected. **f**, **g** Oncospheres and non-spheres were enriched from bladder cancer sample (**f**) or T24 cell line (**g**), followed by immunofluorescence detection of m^6^A modification. DIC, differential interference contrast. Six samples were examined and similar results were obtained, and sample #1 results were shown. *P < 0.05, **P < 0.01, ***P < 0.001, by two-tailed T test. At least three independent experiments were performed and got similar results

To further examine m^6^A modification in bladder TICs, we enriched CD44^+^ bladder TICs and CD44^−^ non-TICs, and detected m^6^A modification. Compared with non-TICs, lower m^6^A modification levels were found in bladder TICs (Fig. 2a), which was confirmed by RNA dot blot and immunofluorescence (Fig. 2b, c).

TICs can survive in sphere formation medium and sphere formation assay emerges as a standard method to enrich bladder TICs. Accordingly, sphere formation assay was performed and spheres were collected to detect m^6^A modification. Compared with non-spheres, lower m^6^A modification levels were found in oncospheres (Fig. 2d). RNA dot blot and immunofluorescence also confirmed the decreased content of m^6^A modification in bladder cancer spheres (Fig. 2e, f). Meanwhile, lower m^6^A modification was also observed in T24 spheres (Fig. 2g). Taken together, m^6^A modification was decreased in bladder TICs.

### Mettl14 was lowly expressed in bladder TICs and accounted for m^6^A suppression

**Fig. 3:**
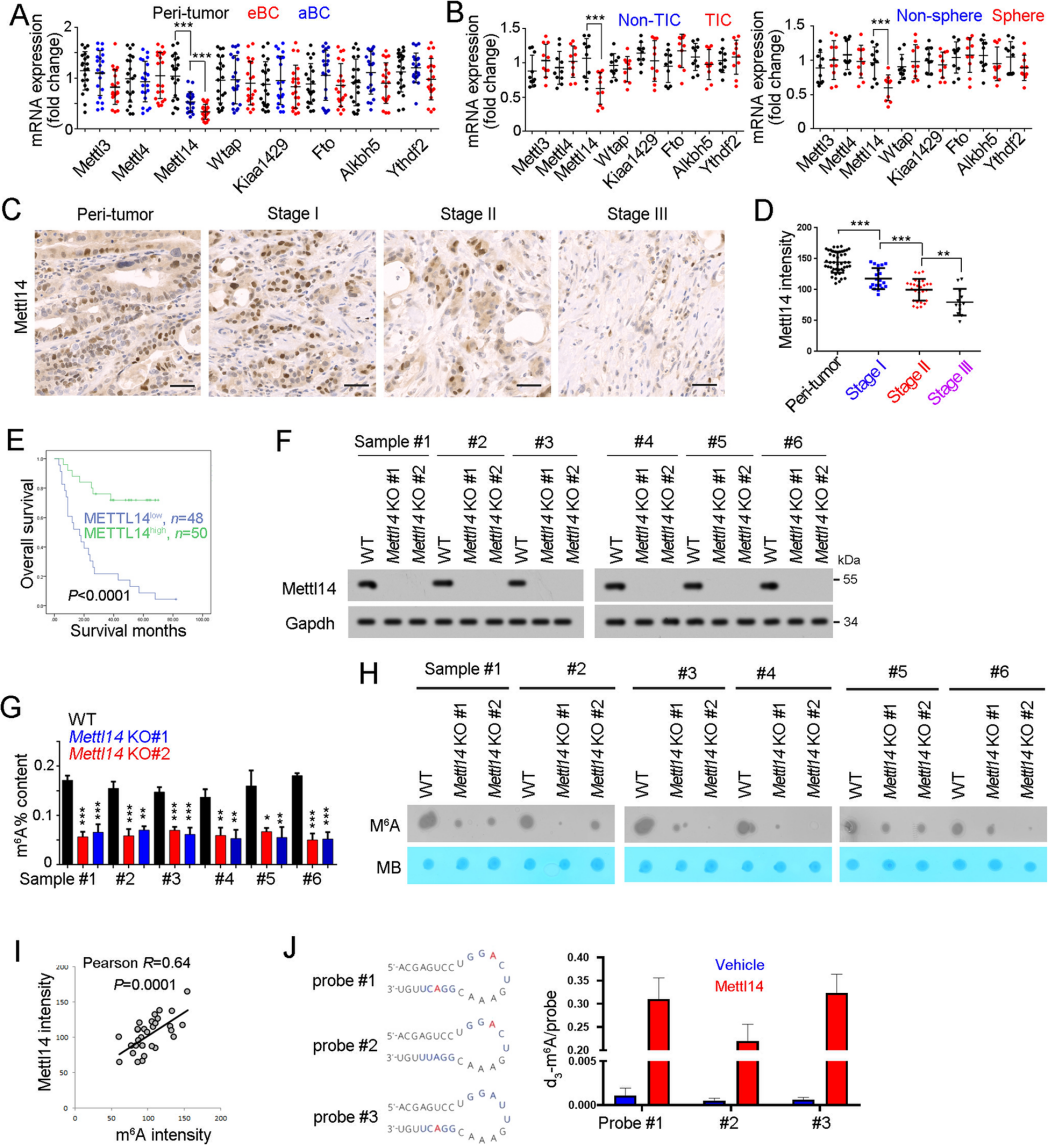
Down-expression of Mettl14 in bladder TICs drove decreased m^6^A modification. **a** Realtime PCR was performed to detect the expression of m^6^A-associated genes in 20 peri-tumors, 20 eBC and 20 aBC samples. **b** 10 bladder cancer samples were used for TIC enrichment and sphere formation, followed by realtime PCR analyses for the expression of indicated genes. **c**, **d** Immunohistochemistry for Mettl14 expression in tissue array containing 46 peri-tumors, 20 stage I, 27 stage II and 12 stage III bladder tumors. Typical images were shown in C and quantitative results of m^6^A modification were shown as scatter diagram (**d**). **e** Bladder cancer patients were grouped into Mettl14^high^ and Mettl14^low^ groups, followed by Kaplan–Meier survival analysis. **f** *Mettl14* knockout bladder cancer cells were generated through CRISPR/Cas9 strategy, and knockout efficiency was confirmed by Western blot. **g** The indicated *Mettl14* knockout cells were used for m^6^A detection. **h** m^6^A content in *Mettl14* knockout cells was detected by RNA dot blot. **i** Correlation of m^6^A modification content and Mettl14 expression. The intensity of m^6^A and Mettl14 was used for. Pearson correlation coefficient (R) and *P*-value analyses. **j** The in vitro RNA N^6^-adenosine methylation activities of Flag-tagged METTL14 were tested using different RNA probes. *P < 0.05, **P < 0.01, ***P < 0.001, by two-tailed T test

Considering the importance of methyltransferases, demethylases and m^6^A readers in m^6^A modification, we then detected the expression profiles of m^6^A-related genes. Among these genes, Mettl14 was lowly expressed in bladder cancer, especially in advanced bladder cancer samples (Fig. 3a). What’s more, lower expression of Mettl14 was detected in bladder TICs and spheres (Fig. 3b). To further examine Mettl14 expression pattern, bladder cancer tissue array was performed and confirmed the lowly expression of Mettl14 in bladder cancer (Fig. 3c). Interestingly, Mettl14 was also lowly expressed in advanced bladder tumors and related to the prognosis of bladder tumor patients (Fig. 3d, e).

To further confirm the role of Mettl14 in m^6^A modification, we generated *Mettl14* knockout cells through CRISPR/Cas9 approach (Fig. 3f). *Mettl14* knockout led to decreased content of m^6^A modification, indicating the critical role of Mettl14 in m^6^A modification (Fig. 3g, h). Moreover, a positive correlation of Mettl14 expression and m^6^A content was observed in bladder tumors (Fig. 3i). What’s more, in vitro RNA N^6^-adenosine methylation assay also confirmed the activity of Mettl14 in RNA m^6^A modification (Fig. 3j). Altogether, Mettl14 was lowly expressed in bladder cancer and accounted for the decreased content of m^6^A modification.

### *Mettl14* knockout drove bladder TIC self-renewal

**Fig. 4:**
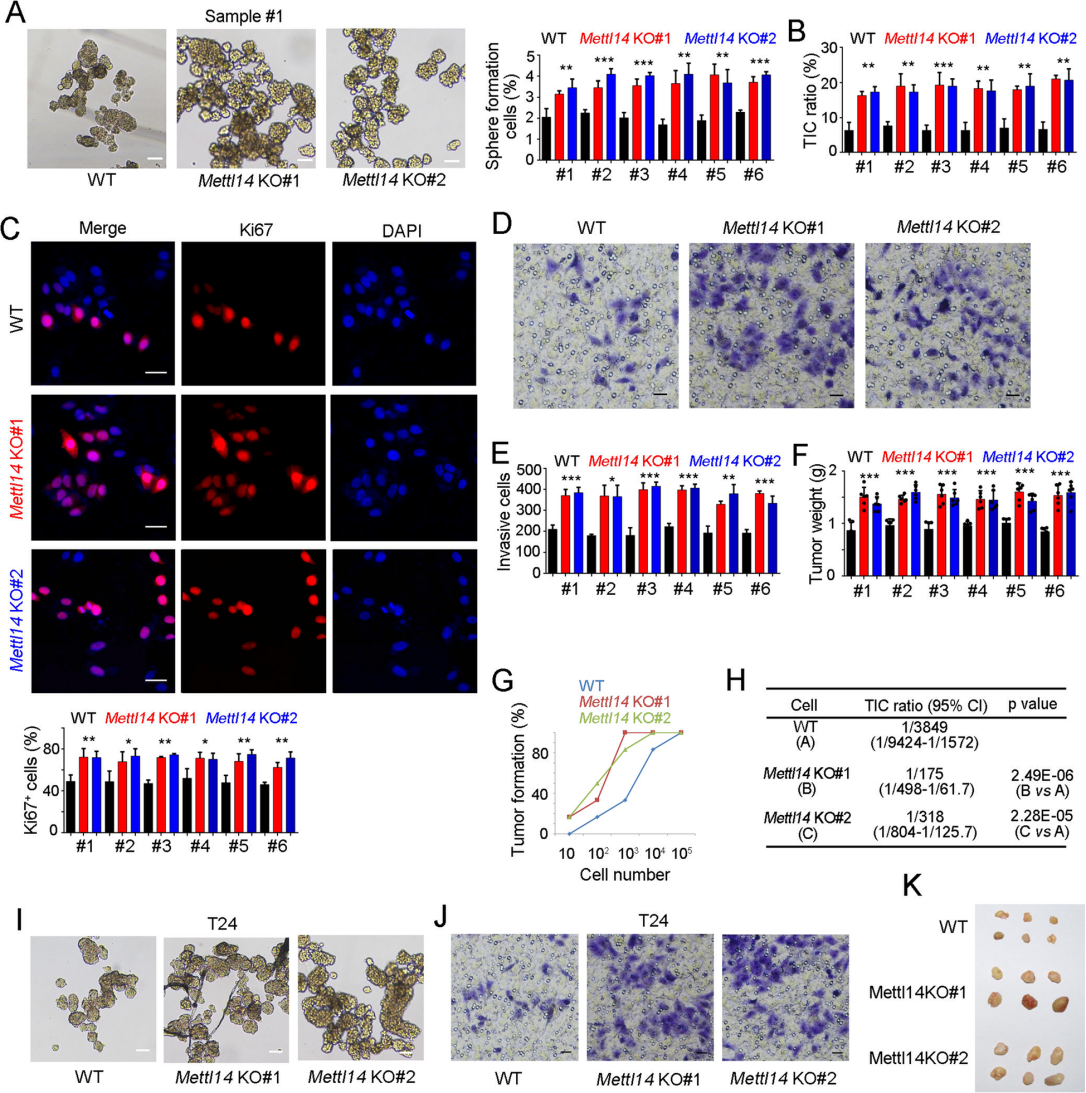
Mettl14 knockout promoted bladder TIC self-renewal. **a** *Mettl14* knockout cells were used for oncosphere formation. Two weeks later, typical images of spheres were taken and sphere-formation ratios were quantitated. **b** The ratios of CD44^+^ bladder TICs in *Mettl14* knockout cells were analyzed by FACS. **c** *Mettl14* knockout and control cells were used for Ki67 staining, and counterstained with DAPI. Typical images were shown in upper panel and Ki67^+^ cell ratios were shown in lower panel. **d**, **e** *Mettl14* knockout and control cells were used for transwell invasion assay. 36 h later, invasive cells were stained with crystal violet for visualization. Typical images were shown in D and cell numbers were counted in **e**. **f** 5 × 10^6^ *Mettl14* knockout cells were subcutaneously injected into BALB/c nude mice, and tumor weight was measured one month later. *n* = 6 for each sample. **g**, **h** 10, 1 × 10^2^, 1 × 10^3^, 1 × 10^4^, and 1 × 10^5^ *Mettl14* knockout and control cells were used for 3 months’ tumor initiation assay. Six BALB/c nude mice were used per sample and the ratios of tumor formation mice were shown (**g**). Bladder TIC ratios were analyzed by extreme limiting dilution analysis (**h**). VS, versus. Six patients were all examined for tumor initiation assay, and only the results of patient #1 were shown. **i** *Mettl14* knockout T24 cells were used for oncosphere formation, and typical images of spheres were taken two weeks later. **j** *Mettl14* knockout T24 cells were used for transwell invasion assay. 36 h later, invasive cells were stained with crystal violet for visualization. **k** *Mettl14* knockout T24 cells were used for tumor propagation assay, and the picture of established tumors was taken. *P < 0.05, **P < 0.01, ***P < 0.001, by two-tailed T test. At least three independent experiments were performed and got similar results

To further explore the role of Mettl14 in bladder tumorigenesis and bladder TICs, we utilized *Mettl14* knockout cells to perform sphere formation assay. *Mettl14* knockout cells showed increased capacity of sphere formation, indicating the inhibitory role of Mettl14 in bladder TIC self-renewal (Fig. 4a). Moreover, *Mettl14* knockout cells contained increased bladder TICs, which confirmed the inhibitory role of Mettl14 in bladder TIC maintenance (Fig. 4b). Cell proliferation was detected by Ki67 staining, and *Mettl14* knockout cells showed enhanced proliferation capacity (Fig. 4c). Bladder TICs account for bladder tumor invasion and metastasis, and thus tumor invasion was also detected. *Mettl14* knockout cells have enhanced invasion capacity, revealing the inhibition of Mettl14 in bladder tumor invasion (Fig. 4d, e). We then detected tumor propagation of *Mettl14* knockout cells through in vivo tumor propagation assay. Increased tumor propagation was found in *Mettl14* knockout cells (Fig. 4f).

To further explore the role of Mettl14 in tumor initiation, *Mettl14* knockout cells were used for tumor initiation assay in vivo. Gradient *Mettl14* knockout cells were injected into BALB/c nude mice for tumor initiation. *Mettl14* deleted cells showed enhanced tumor formation ability (Fig. 4g). Consist with tumor initiation, higher ratios of bladder TICs were also detected in *Mettl14* knockout cells (Fig. 4h). Meanwhile, *Mettl14* deleted T24 cells showed enhanced oncosphere formation capacity (Fig. 4i), enhanced invasion capacity (Fig. 4j) and tumor propagation ability (Fig. 4k). Altogether, *Mettl14* knockout promoted the self-renewal of bladder TICs.

### Mettl14 overexpression inhibited TIC self-renewal

**Fig. 5:**
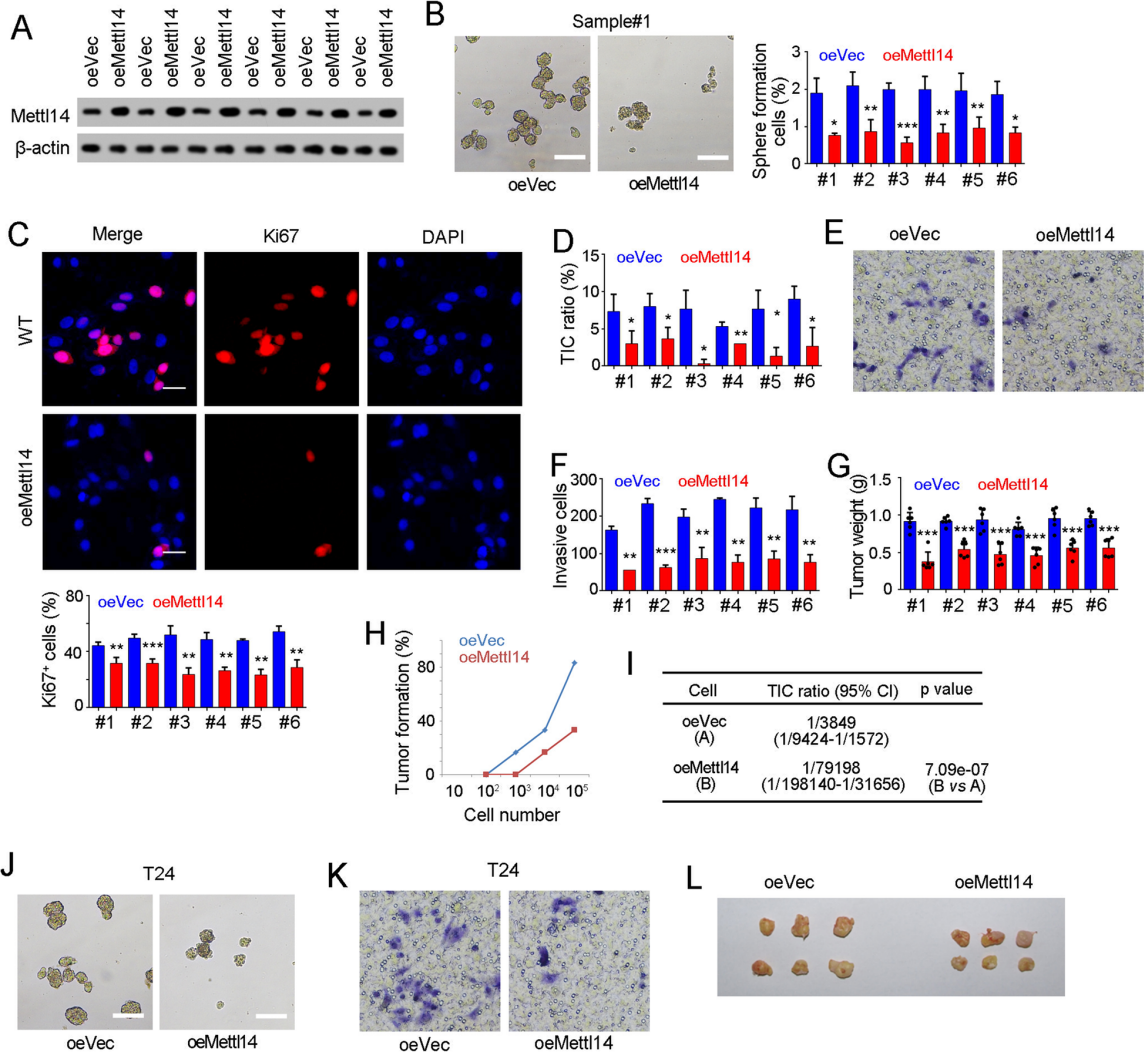
Mettl14 overexpression inhibited bladder TIC self-renewal. **a** Mettl14 overexpressing cells were generated by lentivirus and confirmed by Western blot. oe, overexpression. **b** Mettl14 overexpressing cells were used for oncosphere formation assay. Spheres were shown in left panel and sphere-initiating ratios were shown in right panel. **c** Ki67 immunohistochemistry was performed using Mettl14 overexpressing and control cells. Typical images derived from patient #1 were shown. **d** Mettl14 overexpressing cells were used to detect CD44^+^ bladder TICs by FACS. The ratios of bladder TICs were shown. **e**, **f** Mettl14 overexpressing cells were used for transwell invasion assay. Typical images derived from patient #1 and invasive cell numbers were shown in E and F, respectively. **g** 5 × 10^6^ Mettl14 overexpressing cells were utilized for tumor propagation and tumor weight was detected one month later. **h**, **i** 1 × 10^2^, 1 × 10^3^, 1 × 10^4^ and 1 × 10^5^ Mettl14 overexpressing cells were used for tumor initiation assay. Tumor formation ratios and bladder TIC ratios were shown in H and I, respectively. Six patients were all examined for tumor initiation assay, and only the results of patient #1 were shown. **j**, **k** Mettl14 overexpressing T24 cells were established, and used for sphere formation assay (**j**) and transwell invasion assay (**k**). **l** *Mettl14* overexpressing T24 cells were used for tumor propagation assay, and the picture of established tumors was taken

We then overexpressed Mettl14 in bladder TICs and examined the self-renewal. Mettl14 overexpressing cells were generated through lentivirus and confirmed by Western blot (Fig. 5a). Mettl14 overexpression led to a decrease in sphere formation (Fig. 5b), cell proliferation (Fig. 5c) and TIC ratios (Fig. 5d). Meanwhile, Mettl14 overexpressing cells showed impaired role in tumor invasion (Fig. 5e, f) and propagation (Fig. 5g).

Mettl14 overexpressing cells were also used for tumor-initiating assay, and impaired tumor initiation was observed (Fig. 5h). Decreased ratios of bladder TICs were also calculated through extreme limiting dilution analysis (Fig. 5i). Meanwhile, the inhibitory role of Mettl4 in bladder cancer sphere formation, invasion and tumor propagation was also confirmed in T24 cells (Fig. 5j-l). Altogether, Mettl14 overexpression blocked the self-renewal of bladder TICs.

### Mettl14 targeted Notch1 mRNA stability in bladder TICs

**Fig. 6:**
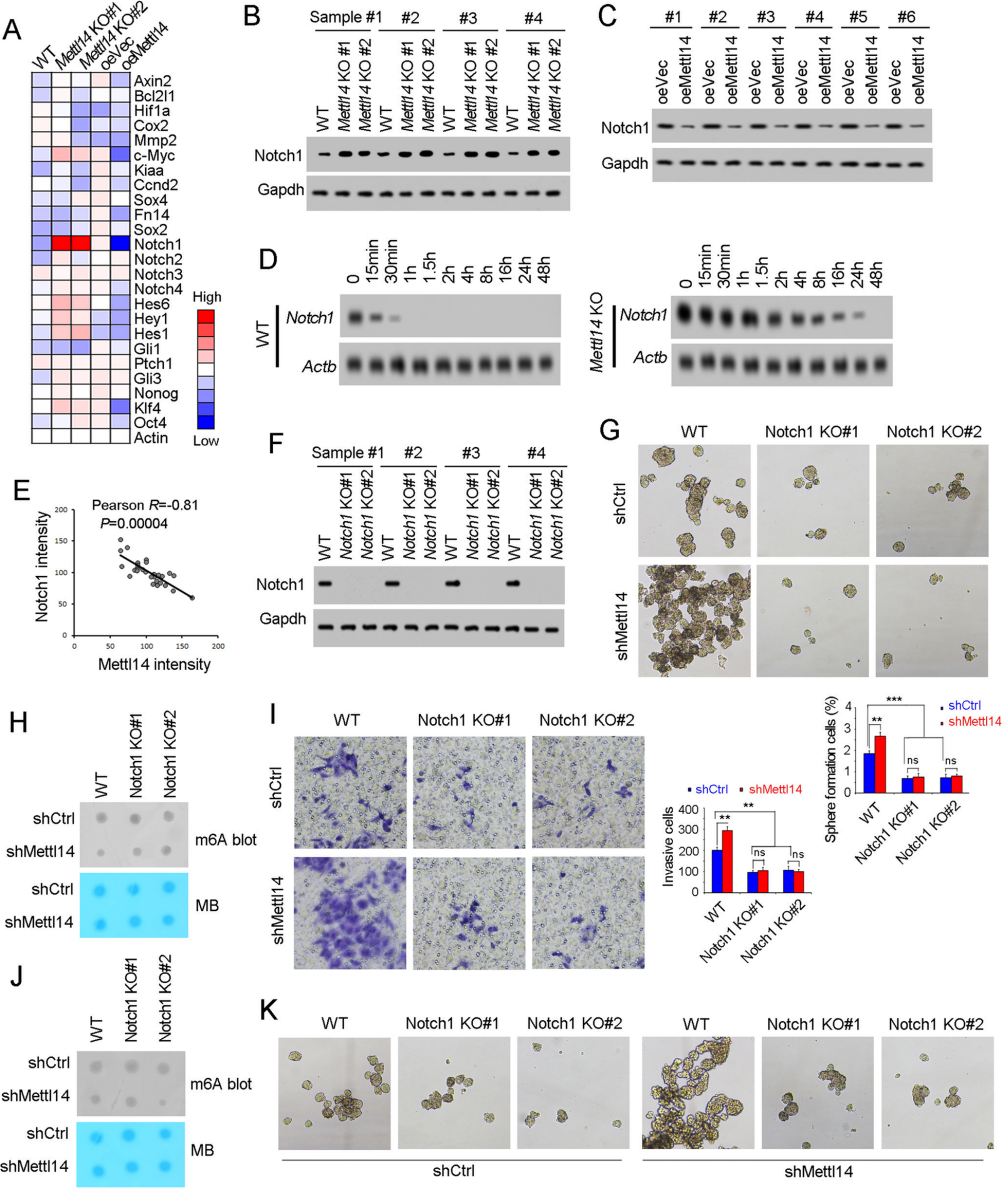
Mettl14 and m^6^A modification inhibited the stability of Notch1. **a** The related genes of Wnt/β-catenin, Notch and Hedeghog signaling were analyzed in *Mettl14* knockout cells and Mettl14 overexpressing cells, and the expression was shown as heatmap. **b**, **c** Notch1 expression levels in *Mettl14* knockout cells (**b**) and Mettl14 overexpressing cells (**c**) were examined by Western blot. Gapdh served as a loading control. **d** *Mettl14* knockout cells were treated with 2 μg/mL actinomycin D, and then Notch1 mRNA levels at the indicated time points were examined by Northern blot. Actb was a loading control. **e** Correlation of Notch1 and Mettl14 expression. The expression levels of Notch1 and Mettl14 were used for analysis. Pearson correlation coefficient (R) and *P*-value were calculated. **f** *Notch1* knockout cells were generated through CRISPR/Cas9 approach and examined by Western blot. **g**, **h**) Sphere formation of *Notch1* knockout cells. For G, typical images were shown in upper panels and calculated numbers were shown in lower panels. For H, spheres were detected for m6A levels, confirming the decreased m6A levels upon Mettl14 knockdown. **i**, **j** Sphere formation of *Notch1* knockout cells. For I, typical images were shown in left panels and calculated numbers were shown in right panels. For J, invasive cells were detected for m6A levels, confirming the decreased m6A levels upon Mettl14 knockdown. **k** Mettl14 was silenced in *Notch1* knockout T24 cells, followed by sphere formation assay. ***P < 0.001; ns, not significant, by two-tailed T test. At least three independent experiments were performed and got similar results

Finally we explored the functional target genes in bladder TICs. We focused on the target genes of three major pathways, including Wnt/β-catenin, Notch and Hedgehog pathways, and found Notch1 was highly expressed in *Mettl14* knockout cells, and lowly expressed in Mettl14 overexpressing cells (Fig. 6a). The inhibitory role of Mettl14 in Notch1 expression was confirmed by Western blot (Fig. 6b, c). Considering the role of m^6^A modification in mRNA stability [18, 19], we examined the stability of Notch1 mRNA in *Mettl14* knockout cells. Interestingly, enhanced stability of Notch1 was observed upon *Mettl14* knockout (Fig. 6d). Moreover, a negative correlation of Mettl14 expression and Notch1 expression was observed in bladder tumors (Fig. 6e).

Although the role of Notch signaling in TICs is well-known, its role in bladder TICs remains elusive. To explore the role of Notch1 in bladder TICs and Mettl14 function, we generated *Notch1* knockout cells through CRISPR/Cas9 approach (Fig. 6f). Compared with control cells, *Notch1* knockout cells showed impaired sphere formation and invasion capacities, revealing the essential role of Notch1 in bladder TICs (Fig. 6g-j). More importantly, in *Notch1* knockout cell, *Mettl14* knockdown showed impaired role for sphere formation and metastasis, indicating the critical role of Notch1 in Mettl14 function (Fig. 6g-j). The essential role of Notch1 in Mettl14 function was confirmed in T24 bladder cancer cell line (Fig. 6k). Altogether, Mettl14 targeted Notch1 that was essential for bladder TICs.

## Discussion

As the most abundant modification in human mRNA, m^6^A modification participates in many physiological and pathological processes [28]. However, its role in bladder tumorigenesis and bladder TICs is unknown. In this work, we discovered the low content of m^6^A modification in bladder tumorigenesis and bladder TICs (shown in Figs. 1 and 2), and identified the role of m^6^A modification and Mettl14 through various functional assays, including sphere formation, transwell invasion assay, gradient tumor initiation assay, tumor propagation assay and Ki67 staining (shown in Figs. 4 and 5). Our work defined Mettl14 as a tumor suppressor gene in bladder and a negative modulator in bladder TICs (shown in Figs. 3, 4 and 5).

The self-renewal of bladder TIC is precisely regulated, and the regulation mechanism is largely unknown. Here, we revealed a novel regulatory axis of bladder TICs. *Mettl14* knockout drove the self-renewal of bladder TICs (shown in Fig. 4), and Mettl14 overexpression inhibited bladder TIC self-renewal (shown in Fig. 5). Mettl14 largely attenuated Notch1 expression, and participated in bladder TICs through Notch1 (shown in Fig. 6). Mettl14-m^6^A-Notch1 pathway plays a critical role in bladder tumorigenesis and bladder TICs.

Wnt/β-catenin, Notch and Hedgehog pathways were the most important signaling pathways in TICs of various tumors [29–32]. We detected the related genes of Wnt/β-catenin, Notch and Hedgehog signaling, and identified Notch1 was a functional target gene of m^6^A modification and Mettl14 (Fig. 6a). Then we generated *Notch1* knockout cell and re-evaluated the role of Mettl14. Impaired Mettl14 role was found upon *Notch1* knockout, indicating Mettl14 mainly target Notch1 to inhibit bladder TIC (Fig. 6). As a common enzyme, there must be many target genes of Mettl14, but here we defined Notch1 was the functional target gene through loss of function assay. We think some other target genes may also involve in other biological processes, but in bladder tumorigenesis and bladder TIC self-renewal, Mettl14 exerted its role mainly through Notch1-dependent manner.

TICs were regulated by various signaling pathways, including Wnt/β-catenin, Notch and Hedgehog pathways. Increasing works revealed the critical role of Wnt/β-catenin and Hedgehog pathway in bladder tumorigenesis and bladder TIC self-renewal. However, the role of Notch signaling in bladder tumorigenesis and TICs remains elusive. Here we generated Notch1 *knockout* cells and revealed an impaired activity of bladder TICs (shown in Fig. 6). Our work defined the oncogenic role of Notch1 in bladder cancer.

The mRNA modification is a complicated process, with various modifications for various RNA molecules. Besides m^6^A, N^1^-methyladenosine, m^5^C and pseudouridine also emerge as critical modulators in various biological processes [33, 34]. m^6^A modification has been identified on mRNA and some non-coding RNA, including microRNA, lncRNA and snoRNA [35]. Increasing evidences reveal that lncRNA emerges as critical modulators in the self-renewal of many kinds of TICs [36–38]. The role of m^6^A and other modifications in non-coding RNAs also need to be further investigated.

## Conclusion

Bladder TICs drive bladder tumorigenesis and metastasis, and their regulation remains largely unknown. Here we revealed the role of *N*^6^-methyladenosine in bladder TIC self-renewal, adding new layers of bladder TIC regulation and *N*^6^-methyladenosine function. As a novel modulator of TICs, Mettl14-*N*^6^-methyladenosine-Notch1 pathway may be a potential target for bladder TIC elimination.

## Data Availability

All data and materials can be provided upon request.

## Abbreviations

CSC: Cancer stem cells
m6A: N6-methyladenosine
TIC: Tumor initiating cells
