# Correction: Mettl14 inhibits bladder TIC self-renewal and bladder tumorigenesis through N6 - methyladenosine of Notch1

**DOI:** 10.1186/s12943-023-01717-x

**Published:** 2023-01-12

**Authors:** Chaohui Gu, Zhiyu Wang, Naichun Zhou, Guanru Li, Yiping Kou, Yang Luo, Yidi Wang, Jinjian Yang, Fengyan Tian

**Affiliations:** 1 Department of Urology and Henan Institute of Urology, Zhengzhou Key Laboratory for Molecular Biology of Urological Tumor Research, The First Affiliated Hospital of Zhengzhou University, Zhengzhou, Henan 450052 People’s Republic of China; 2 Department of Pediatrics, The First Affiliated Hospital of Zhengzhou University, Zhengzhou, Henan 450052 People’s Republic of China

**Correction: Mol Cancer 18, 168 (2019)**

**https://doi.org/10.1186/s12943-019-1084-1**

Following the publication of the original paper [1], we have found several inadvertent errors in the figures recently. Those errors are as following:

The images for sphere formation assays in Fig. 6G (WT/shCtrl group), and dot blot assays in Fig. 2B and E were misused. After a self-investigation and carefully check of the archived images and raw data of this study, we found these errors happened inadvertently due to these images from different groups or different times were saved in a corresponded folder during the stage of saving the images. Besides, there is a mistake in Fig. 6D (WT and Mettl14 KO group). We incorrectly marked the superfluous time point “1.5 h” on top of “Notch1” northern blot images due to our carelessness. The corrected version of Fig. 6G&D and Fig. 2B&E have been provided. We sincerely apologize to the editor, reviewers and readers for the errors and any confusion it may have caused.

**Fig. 2:**
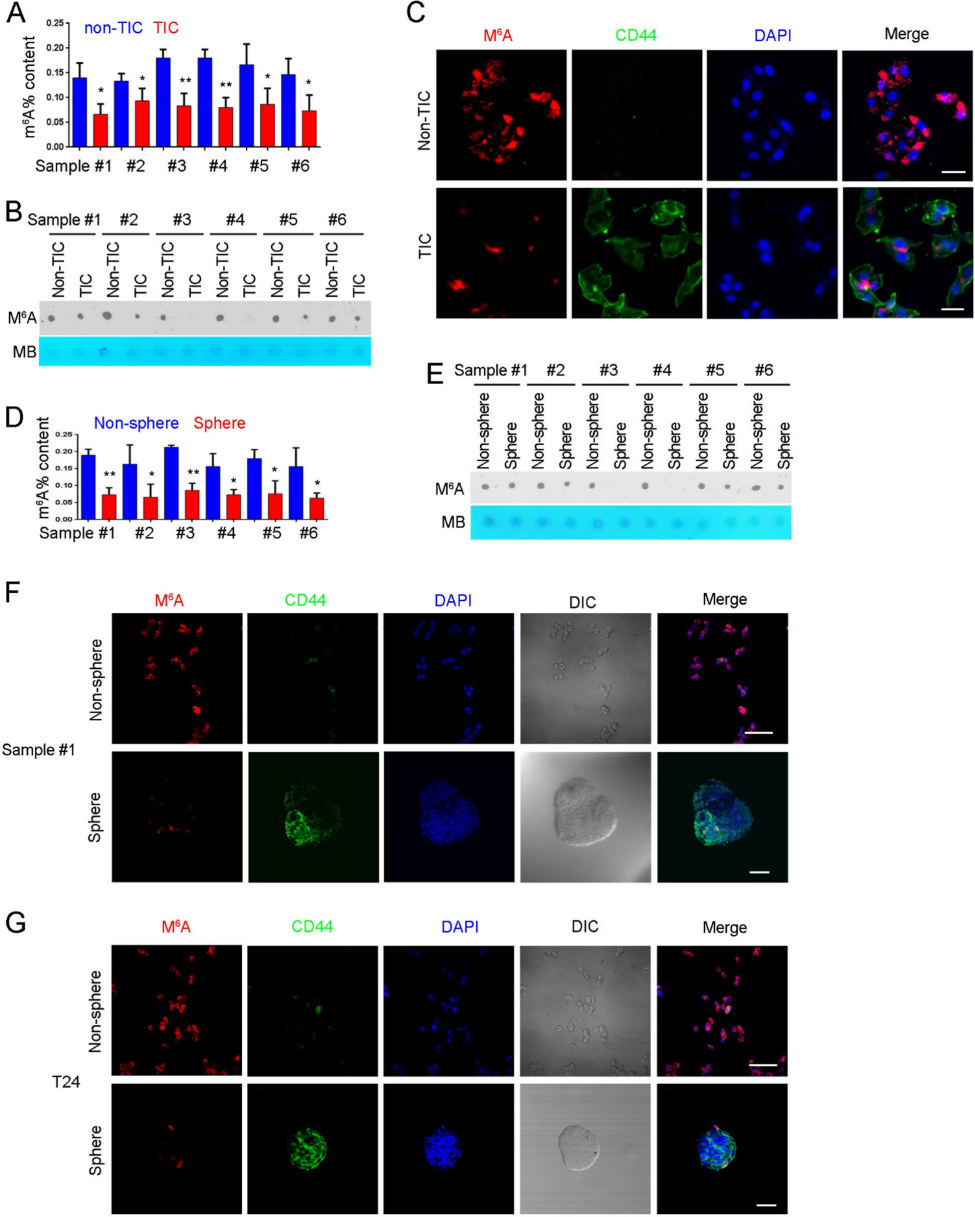
M^6^A modification was reduced in bladder TICs. **A** Bladder TICs and non-TICs were sorted by FACS with CD44 antibody, and mRNA was extracted for m^6^A detection. Six bladder tumors were used for TIC enrichment and subsequent m^6^A detection. **B** m^6^A RNA dot blot in bladder TICs and non-TICs. RNA extracted from bladder TICs and non-TICs was examined for m^6^A modification. Six samples were examined and got similar results. **C** FACS enriched TICs and non-TICs were stained with m^6^A and CD44 antibodies, and visualized by confocal microscopy. **D** Sphere formation was performed, followed by m^6^A detection using spheres and non-spheres. **E** Spheres and non-spheres were generated from bladder primary cells and m^6^A modification was detected. **F**, **G** Oncospheres and non-spheres were enriched from bladder cancer sample (**F**) or T24 cell line (**G**), followed by immunofluorescence detection of m^6^A modification. DIC, differential interference contrast. Six samples were examined and similar results were obtained, and sample #1 results were shown. *P < 0.05, **P < 0.01, ***P < 0.001, by two-tailed T test. At least threeindependent experiments were performed and got similar results

**Fig. 6:**
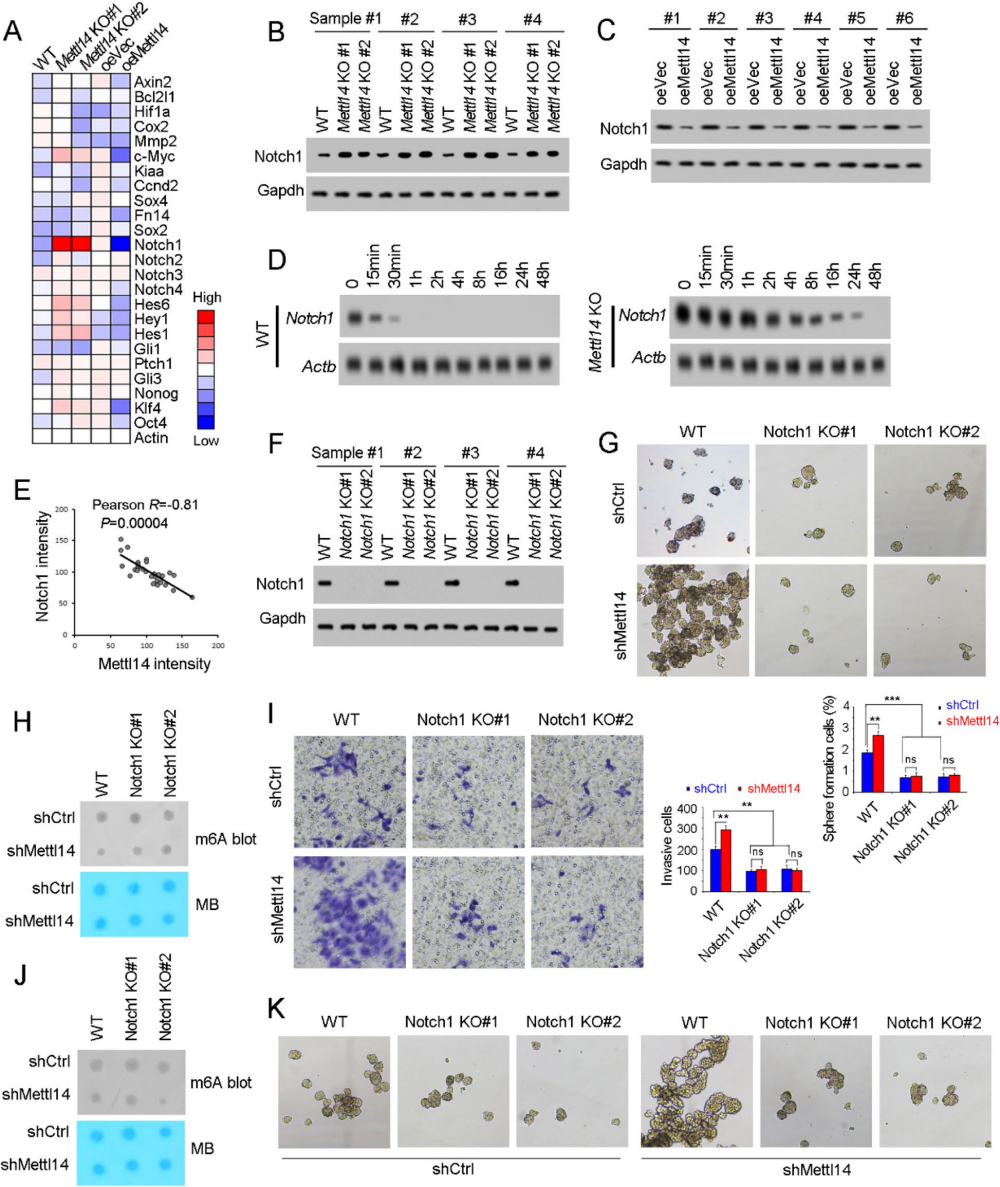
Mettl14 and m^6^A modification inhibited the stability of Notch1. **A** The related genes of Wnt/β-catenin, Notch and Hedeghog signaling were analyzed in *Mettl14* knockout cells and Mettl14 overexpressing cells, and the expression was shown as heatmap. **B**, **C** Notch1 expression levels in *Mettl14* knockout cells (**B**) and Mettl14 overexpressing cells (**C**) were examined by Western blot. Gapdh served as a loading control. **D** *Mettl14* knockout cells were treated with 2 μg/mL actinomycin D, and then Notch1 mRNA levels at the indicated time points were examined by Northern blot. Actb was a loading control. **E** Correlation of Notch1 and Mettl14 expression. The expression levels of Notch1 and Mettl14 were used for analysis. Pearson correlation coefficient (R) and P-value were calculated. **F** *Notch1* knockout cells were generated through CRISPR/Cas9 approach and examined by Western blot. **G**, **H** Sphere formation of *Notch1* knockout cells. For G, typical images were shown in upper panels and calculated numbers were shown in lower panels. For H, spheres were detected for m^6^A levels, confirming the decreased m6A levels upon Mettl14 knockdown. **I**, **J** Sphere formation of *Notch1* knockout cells. For I, typical images were shown in left panels and calculated numbers were shown in right panels. For J, invasive cells weredetected for m6A levels, confirming the decreased m6A levels upon Mettl14 knockdown. **K** Mettl14 was silenced in *Notch1* knockout T24 cells, followed by sphere formation assay. ***P < 0.001; ns, not significant, by two-tailed T test. At least three independent experiments were performed and got similar results
